# Survival and Control of *Campylobacter* in Poultry Production Environment

**DOI:** 10.3389/fcimb.2020.615049

**Published:** 2021-01-29

**Authors:** Mohammed J. Hakeem, Xiaonan Lu

**Affiliations:** ^1^ Food, Nutrition and Health Program, Faculty of Land and Food Systems, The University of British Columbia, Vancouver, BC, Canada; ^2^ Department of Food Science and Human Nutrition, College of Food and Agriculture Sciences, King Saud University, Riyadh, Saudi Arabia; ^3^ Department of Food Science and Agricultural Chemistry, Faculty of Agricultural and Environmental Sciences, McGill University, Ste Anne de Bellevue, QC, Canada

**Keywords:** *Campylobacter*, survival, control, poultry-processing plants, poultry farms

## Abstract

*Campylobacter* species are Gram-negative, motile, and non–spore-forming bacteria with a unique helical shape that changes to filamentous or coccoid as an adaptive response to environmental stresses. The relatively small genome (1.6 Mbp) of *Campylobacter* with unique cellular and molecular physiology is only understood to a limited extent. The overall strict requirement of this fastidious microorganism to be either isolated or cultivated in the laboratory settings make itself to appear as a weak survivor and/or an easy target to be inactivated in the surrounding environment of poultry farms, such as soil, water source, dust, surfaces and air. The survival of this obligate microaerobic bacterium from poultry farms to slaughterhouses and the final poultry products indicates that *Campylobacter* has several adaptive responses and/or environmental niches throughout the poultry production chain. Many of these adaptive responses remain puzzles. No single control method is yet known to fully address *Campylobacter* contamination in the poultry industry and new intervention strategies are required. The aim of this review article is to discuss the transmission, survival, and adaptation of *Campylobacter* species in the poultry production environments. Some approved and novel control methods against *Campylobacter* species throughout the poultry production chain will also be discussed.

## Introduction

The name of *Campylobacter* [kam′′pə-lo-bak′tər] originally came from the ancient Greek meaning curved rod where kampylos means curved and baktron means rod. However, the unique shape of *Campylobacter* looks more like a spiral or helical one rather than a curved rod shape. *Campylobacter* can change its shape into filamentous or coccoid to adapt to the stressful conditions (Gaynor et al., 2005; Tresse et al., 2017). It was first isolated from a sheep abortion case and classified as a *Vibrio-*like bacterium (McFadyean and Stockman, 1913; Skirrow, 2006) and then renamed as *Campylobacter* after showing a clear different taxonomy profile from the *Vibrio* species. *Campylobacter* bacteria are very diverse microorganisms not only on the species levels but also on the subspecies and strain levels (Gaynor et al., 2005; Vidal et al., 2016). Diversity includes differences in genetic and phenotypic characteristics as well as growth requirement, which may explain their presence in different hosts or ecological niches including different poultry and wild birds. Some *Campylobacter* species are flagellated with a single polar flagellum or bipolar flagella (*e.g*., *C. jejuni*, *C. coli C. concisus* and *C. showae*), while fewer species (*e.g*., *C. hominis* and *C. ureolyticus*) have no flagellum (Man, 2011).

Emerging *Campylobacter* bacteria are species that have been identified recently to cause illnesses (Kaakoush et al., 2015). They include *C. concisus, C. curvus, C. fetus, C. gracilis, C. mucosalis, C. pinnipediorum, C. rectus, C. showae, C. sputorum, C. lari, C. ureolyticus, C. upsaliensis*, and *C. volucris*. The clinical importance and pathogenicity of emerging *Campylobacter* species have been reviewed (Man, 2011; Costa and Iraola, 2019). Available evidence showed that they could attach and invade human epithelial cells, alter intestinal barrier integrity, avoid host immune response, secrete toxins and invade macrophages. In contrast, the actual contribution of emerging *Campylobacter* species to campylobacteriosis is still not clear because available cultivation methods including hydrogen-enhanced microaerobic and anaerobic conditions failed to successfully grow these microbes under the laboratory condition (Kaakoush et al., 2015). This is due to several reasons including the slow growing nature of some fastidious species or individual strains, growth inhibition by antibiotics added in selective media, limited hydrogen source, presence of competitive microorganisms, and/or difficulties in identifying some *Campylobacter* species due to their morphological diversities. Nevertheless, hydrogen enhancement (generally 3–7%) in the microaerobic condition improved the detection of *C. concisus* from 0.03% to 1.92% (Casanova et al., 2015). Symptoms of *C. concisus* infections and other *Campylobacter* bacterial infections are usually milder than that with C. jejuni and *C. coli*. However, emerging *Campylobacter* species are also important and require better isolation techniques for their detection and diagnosis. A previous report showed that infections of *C. concisus* and *C. fetus* were more common than infections of *C. jejuni* and *C. coli* in the elderly (68.4 years old) than young adults of 28.6 years old on average (Bessède et al., 2014). In conclusion, although *C. jejuni* and *C. coli* remain the leading cause of campylobacteriosis, more effective detection methods are required for a better understanding of how emerging *Campylobacter* bacteria evolve in the environment, transmit to agri-food systems, and contribute to campylobacteriosis.

Available evidence suggests that campylobacteriosis incidence has been rising in both developed and developing countries in the recent years (Kaakoush et al., 2015). The size of *Campylobacter* outbreaks in different countries ranged from 10 to 100 cases between 2007 and 2013 (Kaakoush et al., 2015). Poultry and untreated water were the most reported sources of *Campylobacter* outbreaks. The number of *Campylobacter* cases in different countries within the same region can vary significantly. This is not only due to the unreported cases but also limited sensitivity of detection methods, population size and composition, variation in public health standards, intervention strategies, surveillance systems, food safety practices, and the prevalence of *Campylobacter* in natural reservoirs in different regions. The epidemiological data from Asia, Africa, and the Middle East shows that *Campylobacter* infection is prevalent in this region although the data is incomplete. The total number of *Campylobacter* infections in Canada was estimated to be about 145,350 cases per year (Thomas et al., 2013). British Columbia (BC) had an annual *Campylobacter* infection rate of 37.74 cases per 100,000 people (1,818 cases) in 2017 (BC Center for Disease Control, 2017). In comparison, Japan had a rate of 1,512 cases per 100,000 people (Kubota et al., 2011) and New Zealand had a rate of 161.5 per 100,000 people (Sears et al., 2011) within the last decade. In USA, the surveillance system, new regulations, and control strategies have contributed to the decline of several foodborne pathogens including *Salmonella*, *Listeria*, and *E. coli* O157:H7 from 2006 to 2014, but not *Campylobacter* and *Vibrio* (Crim et al., 2014). Altogether, both individual cases and outbreaks of campylobacteriosis are generally prevalent around the world.

Several risk factors that can lead to *Campylobacter* infections include traveling or person-to-person transmission, contact with animals, and consumption of contaminated food or water. Meta-analysis data suggest that international and domestic traveling was the most critical risk factor of *Campylobacter* infections, followed by the consumption of uncooked chicken meat, environmental exposure, and direct contact with the farm animals. A Canadian report showed that campylobacteriosis was responsible for the highest number of causes of travel-related diseases [123/446 cases (27.57%)] from 2005 to 2009 (Ravel et al., 2010). In addition, overlapping exists between risk factors. For example, travel-related diseases are frequently linked to the consumption of contaminated foods (Kaakoush et al., 2015). Although traveling abroad contributes to the overall *Campylobacter* transmission, the spread of antibiotic-resistant *Campylobacter* strains between countries and continents through international agri-food trade is also a considerable public health concern (Mughini-Gras et al., 2014).

## The Unique Physiology of *Campylobacter*

*Campylobacter* species are not only unique in their shape, but they also have a relatively small genome with unique cellular and molecular physiology compared to other foodborne pathogens. The first whole-genome sequencing analysis of *C. jejuni* (NCTC11168 strain) showed that the genome (1.6 Mbp) has uniquely a limited number of repeated sequences and no insertion or phage associated regions (Parkhill et al., 2000). Other reports showed that *C. jejuni* lacks the regulator *rpoS* (starvation/stationary phase sigma factor) and their stationary-phase cultures are ununiformed dynamic populations unlike most of other bacteria (Kelly et al., 2001). This could be a survival strategy that *C. jejuni* uses to reduce its starvation stress during the stationary phase at least in some strains. Although the existence of stationary phase in *C. jejuni* is elusive, a transition from exponential to stationary phase was observed in *C. jejuni* populations with a number of changes in the transcriptomic and proteomic profiles between the two phases (Turonova et al., 2017). These data also suggest that the pleiotropic regulator *cosR* gene acts as a negative autoregulator and is alternative to *rpoS* gene in *C. jejuni* during the stationary phase of growth. In addition, *C. jejuni* is an asaccharolytic bacterium (*i.e.*, unable to break down carbohydrate for energy) due to the absence of some key glycolytic enzymes [*e.g.*, glucokinase (GIK) and phosphofructokinase (Pfk)] that involved in the functional Embden-Meyerhof-Parnas glycolysis pathway (Tresse et al., 2017). *Campylobacter* is also a chemo-organotrophic bacterium that oxidizes the chemical bonds in amino acids or intermediate molecules of tricarboxylic acid (Krebs) cycle as their energy and carbon source. Moreover, *C. jejuni* uses gluconeogenesis fueled by amino acids to generate glucose from non-carbohydrate sources. The Entner-Doudoroff (ED) pathway is used in bacteria for synthesizing pyruvate from extracellular glucose. A complete group of genes encoding ED pathway was identified in some rare *C. jejuni* and *C. coli* isolates (Vegge et al., 2016). Interestingly, this gene set increased the survival and biofilm formation in *Campylobacter*. Altogether, *C. jejuni* lacks many important stress response genes, but has developed different mechanisms to adapt to and survive in the new environmental and/or under stress conditions.

*Campylobacter* species have many unique growth requirements that can limit but not eliminate their prevalence outside warm-blooded hosts in foods and/or food environments. Most *Campylobacter* bacteria grow optimally at either 42**°**C (chicken body temperature) or 37**°**C (human body temperature), but none of them can grow below 30**°**C (Park, 1996). The growth rate of most other bacteria reduces gradually near their minimum growth temperature unlike *Campylobacter* that suddenly stops to grow below 30**°**C (Hazeleger et al., 1998). No growth adaptation of *C. jejuni* was observed below 30**°**C. This raises the question of how different the metabolic activity of *Campylobacter* is below and above the minimum growth temperature. This question will be answered below according to several reports about the survival of *Campylobacter* in food and food-related conditions. Moreover, *Campylobacter* is unable to survive under the ambient oxygen level due to several combined reasons (Mace et al., 2015). These include (i) limited tolerance against reactive oxygen species (ROS), (ii) incompetence of producing adequate antioxidant enzymes, (iii) low respiratory rate, and (iv) presence of oxygen-labile essential enzymes (Velayudhan et al., 2004). A few enzymes present in *Campylobacter* are believed to play a critical role in protecting the cells from oxygen tension. These include catalase, glutathione reductase, glutathione synthetase, peroxidase, and superoxide dismutase (Keener et al., 2004).

## Human Infections

*Campylobacter* is documented in 2019 to be the leading foodborne pathogen associated with the consumption of animal-source food products worldwide (Li et al., 2019). Classical symptoms of *Campylobacter* infections (called campylobacteriosis) include fever, severe watery or bloody diarrhea, cramps, and weight loss for 6 days on average in humans (Véron and Chatelain, 1973; Kaakoush et al., 2015). Most infections are self-limiting and do not require medical therapy other than hydration and electrolyte balance (Acheson and Allos, 2001). Antibiotic treatment is only applied either in severe cases or to immunocompromised individuals. *C. jejuni* and *C. coli* are the major causes of campylobacteriosis in humans (Kaakoush et al., 2015). Several studies showed that infections of both *C. jejuni* and *C. coli* occur more frequently during the summer than other seasons (Nielsen et al., 2013; Bessède et al., 2014). *C. jejuni* infection is greater than *C. coli* in many countries, but *C. coli* is also an important species and reported to be the second most contributor to campylobacteriosis after *C. jejuni*. In fact, a comparison study of patients infected with either *C. jejuni* or *C. coli* showed that slightly older patients (34.6 compared to 27.5 years old) have a greater risk of being infected with *C. coli* than *C. jejuni* (Bessède et al., 2014). Campylobacteriosis has also been linked to a range of gastrointestinal conditions, such as inflammatory bowel diseases (IBD), periodontitis, esophageal disease, functional gastrointestinal disorders, celiac disease, and colon cancer in humans (Véron and Chatelain, 1973; Kaakoush et al., 2015). *C. jejuni* infections may lead to autoimmune disorders known as Guillain-Barré syndrome (GBS) and Miller Fisher syndrome. According to an infection study of 111 volunteers, *C. jejuni* dosage correlated with colonization rate, but not with the development of illnesses (Black et al., 1988). The infectious dose to develop campylobacteriosis varied depending on immunity and health status of the individuals. Only 800 *Campylobacter* cells were able to cause diarrhea to some volunteers, while other data showed that campylobacteriosis was developed with a dose as low as 360 cells (Hara-Kudo and Takatori, 2011). Several genes, proteins and components of *C. jejuni* are involved in different virulence factors (**Table 1**).

**Table 1:** Examples of some important virulence factors and their roles in *C. jejuni*.

Virulence factors	*C. jejuni* gene, protein, or component	Role	References
**Stress response**	*CosR* *GroESL* DnaJ Lon protease RacR-RacS	Oxidative stress response regulator Heat shock operon Heat shock protein Heat shock protein Regulate temperature during growth and colonization	(Hwang et al., 2011) (Thies et al., 1999) (Konkel et al., 1998) (Thies et al., 1999) (Brás et al., 1999)
**Motility and chemotaxis**	fliA ropN FlgR,S CheA, B, R, W, V, Y CheY	Flagellar (sigma 28) → regulates the transcription of flagellar genes Flagellar (sigma 54) → regulates the transcription of flagellar genes Regulate the flagellum protein biosynthesis Chemotaxis signal transduction (Che) proteins network. Response regulator used for flagellar rotation	(Jagannathan et al., 2001) (Jagannathan et al., 2001) (Hendrixson, 2006) (Chandrashekhar et al., 2017) (Yao et al., 1997)
**Adhesion**	RacR-RacS LOS CadF	Persistent colonization of the gut. Involved in adherence and display molecular mimicry of neuronal ganglioside → Guillain-Barré syndrome Fibronectin-binding outer membrane protein	(Van der Stel et al., 2015) (Young et al., 2007) (Konkel et al., 1997)
**Invasion**	Flagellum LOS CPS Cia	Non-flagellated mutants are less invasive. Lipopolysaccharide → Involved in invasion Capsular polysaccharide → Involved in invasion Invasive antigens	(Konkel et al., 1999b) (Grant et al., 1993) (Karlyshev and Wren, 2001) (Rivera-Amill et al., 2001)
**Secretion**	pVir CiaB	Plasmid found in some *C. jejuni* isolates led to type IV secretion system. Play role in invasion and type III secretion system	(Bacon et al., 2002) (Konkel et al., 1999a; Konkel et al., 1999b)
**Toxins**	CdtA, B, C (Cytolethal distending toxin)	Cell distension, cell cycle block and DNA damage → Cell death.	(Lara-Tejero and Galán, 2001)

## Survival in Food and Food-Related Conditions

*Campylobacter* is sensitive to food and food processing-related stresses. It is more sensitive to heat treatment compared to other foodborne pathogens. For example, the *D*-value of *E. coli* is five times higher than that of *C. jejuni* at 55**°**C (Rusin et al., 1997). Simply freezing at −15**°**C could reduce *C. jejuni* count by 3 log CFU/g in ground beef (Stern and Kotula, 1982). Desiccation at room temperature inactivated *Campylobacter* within a few days (Doyle and Roman, 1982b). *Campylobacter* cannot survive for a long period of time on food contact surfaces, such as cutting boards, countertops, equipment or kitchen utensils. In contrast, *Campylobacter* can remain viable on fresh foods, such as ground beef (Stern and Kotula, 1982), fresh produce (Kärenlampi and Hänninen, 2004), fresh chicken (Blankenship and Craven, 1982), and milk (Doyle and Roman, 1982b) during the entire shelf life up to 3 weeks. In addition, the combination of these wet and cold refrigeration conditions of fresh foods assists *Campylobacter* in surviving on dry surfaces for a few weeks instead of a few days (Doyle and Roman, 1982a; Stern and Kotula, 1982).

**Figure 1:**
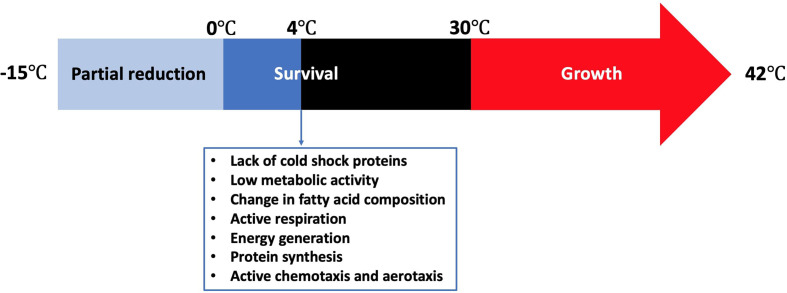
Temperature range for the survival of *Campylobacter* and its stress response at 4°C.

Refrigeration is one of the most common food preservation methods either used alone or in combination with other antimicrobial strategies or food preservation methods such as the addition of preservatives, irradiation, or modification of atmosphere. *Campylobacter* grows in a limited temperature range compared to other food microorganisms (**Figure 1**). The growth rates of the majority of microorganisms drop to the minimum or stop at refrigeration temperatures. However, fewer pathogenic and spoilage bacteria can grow from a few cells to a large number (*e.g*., psychrotrophic bacteria) and cause serious food poisonings (Chan and Wiedmann, 2008) or spoilage recall incidents that can be associated with food loss and negative impact on the economy (Pothakos et al., 2014). *Pseudomonas* species (Chouliara et al., 2007; Zhang et al., 2012; Al-Nehlawi et al., 2013), lactic acid bacteria (Chouliara et al., 2007; Doulgeraki et al., 2012; Zhang et al., 2012), and *Brochothrix thermosphacta* (Chouliara et al., 2007; Zhang et al., 2012) are considered as the most problematic spoilage psychrotrophic bacteria in poultry meat. In contrast, *Campylobacter* and *Salmonella* are the most causes of human gastroenteritis due to poultry meat consumption (Rouger et al., 2017). *Campylobacter* in poultry is ranked as the leading pathogen-food combination to cause health risks and negatively impacts the economy (Batz et al., 2012). Kaakoush and others reported that poultry consumption was the most cause of campylobacteriosis outbreaks between 2007 and 2013 (Kaakoush et al., 2015). A more recent report showed that 28 campylobacteriosis outbreaks were linked to the consumption of chicken livers in USA between 2000 and 2016 (Lanier et al., 2018). Up to 90% of commercially available chicken meat in different regions has been identified to be contaminated by *Campylobacter* at ~log 4 CFU/carcass (Willis and Murray, 1997; Jorgensen et al., 2002; Walker et al., 2019).

Cold stress response of *Campylobacter* is significantly different from other common foodborne pathogens. Although *Campylobacter* lacks cold shock proteins, this microbe can still be active during the shelf life of different refrigerated foods or during the winter season in the agro-ecosystem (Murphy et al., 2006). Hazeleger and others compared the changes in fatty acid composition of the membrane of coccoid-shaped *Campylobacter* cells with that of the spiral-shaped cells incubated at 4**°**C (Hazeleger et al., 1995). The change in the fatty acid composition in both groups was similar. In contrast, a significant change in the composition of fatty acids occurred when the cells were incubated either at 12**°**C or 25**°**C. This included a significant increase in the percentage of 16:0 and 18:0 fatty acids and a significant decline in the percentage of 14:0, 16:1 and 19:0 fatty acids. The same group reported in another study that the vital processes of *C. jejuni* including cellular respiration, catalase activity, energy generation, and protein synthesis were still be functional at 4**°**C, which was far below the minimum growth temperature at 30**°**C (Hazeleger et al., 1998). The total amount of ATP (*i.e*., produced + consumed) as indicated by the respiration rate at 4**°**C was only 5% of that at 40**°**C, suggesting that *C. jejuni* has a relatively low metabolic activity at low temperatures. However, the concentration of the produced ATP at 4**°**C was almost 50% of that at 40**°**C. Physiological functions such as chemotaxis and aerotaxis were similarly observed at 4, 20, and 40**°**C, indicating that *C. jejuni* could normally move toward substrates even below 30**°**C. The effect of cold exposure (*i.e*., 6**°**C for 24 h) on the thermal tolerance (*i.e*., 56**°**C) of *C. jejuni* was compared with that of *E. coli* K-12 (Hughes et al., 2009). *C. jejuni* was more tolerant than *E. coli* K-12 to thermal treatment as the ratio of the unsaturated to saturated fatty acids did not change after cold exposure, which was different from that of *E. coli* K-12. In conclusion, *Campylobacter* not only remains viable at low temperatures but also maintains sufficient metabolic activity to survive and move to the favorable places even in the absence of cold shock proteins.

## *Campylobacter* in Poultry Farms

### Transmission into Poultry Farms

Poultry has been considered as the major source of food-related transmission of *Campylobacter* species to humans since the early years of poultry industry (Skirrow, 1977). *C. jejuni* is a common commensal microorganism in chicken microbiome (Hendrixson and DiRita, 2004; Awad et al., 2016; Ijaz et al., 2018). Poultry is also a reservoir of other *Campylobacter* species including *C. lari*, *C. upsaliensis*, and *C. concisus* (Kaakoush et al., 2014). This bacterium usually transmits horizontally from different environmental sources to flocks (Sahin et al., 2002; Kaakoush et al., 2015). It was reported that *Campylobacter* species are usually abundant in the surrounding environment of poultry farms, such as soil, water source, dust, surfaces and air (Ellis-Iversen et al., 2012). Animal feed and/or drinking water can transmit *Campylobacter* from the environment to poultry farms. Farmers and farm visitors who carry *Campylobacter* can also transmit this microbe to poultry farms. Several studies isolated *Campylobacter* from wild bird feces around poultry houses, suggesting that wild birds contributed to the transmission of this microbe into the poultry houses (Craven et al., 2000; Hiett et al., 2002). For example, a molecular subtype analysis showed that 12 *Campylobacter* strains isolated from the broiler flocks were closely related to a strain isolated from wild bird feces identified in the same farm environment (Hiett et al., 2002).

Other organisms including flies, insects, amoebae, yeasts and molds have been found to be also important routes of horizontal transmission of *Campylobacter* into poultry houses (Axelsson-Olsson et al., 2005; Newell et al., 2011). The presence of *Campylobacter* cells with amoebae, yeasts and molds allow them to survive longer. A lesser mealworm beetle and their larvae (*Alphitobius diaperunus*) were identified as important carriers of *C. jejuni* in the poultry facilities. They could transmit *C. jejuni* not only within batches but also cross-contaminate flocks in the successive rearing cycles (Hazeleger et al., 2008). In addition, microbial eukaryotes may act as a reservoir of *Campylobacter* in the environment. For example, numerous *C. jejuni* strains are able to invade, replicate, and remain viable inside an amoeba host (*i.e., Acanthamoeba polyphaga*) (Axelsson-Olsson et al., 2005). Since eukaryotes are usually prevalent in both drinking water systems and microbial biofilms on farms (Snelling et al., 2006), it is highly possible that infected eukaryotes contribute to *C. jejuni* transmission to poultry infrastructure.

There has been a long controversy about whether *Campylobacter* can be transmitted vertically from one generation of poultry to the other (Cox et al., 2012). One study including 60,000 progeny parent breeders identified a lack of evidence for vertical transmission of *Campylobacter* to chickens (Callicott et al., 2006). All chickens used in the study were hatched from eggs of *Campylobacter*-colonized grandparent flocks. However, egg passage can lead to the transmission of fecal bacteria including *Campylobacter* and subsequently contaminate the shell, shell membrane, and albumen of newly laid and fertile eggs (Cox et al., 2012). This can lead to *Campylobacter* ingestion after the chicks emerge from their eggs, colonization and spread of *Campylobacter* in poultry houses. In contrast, vertical transmission is well-established in *Salmonella* as they contaminate the egg within the reproductive tract before the shell is formed or penetrate the eggshell and invade the yolk of the post-lay egg (Gast and Beard, 1990; Miyamoto et al., 1997; Yang et al., 2001). In addition, *Salmonella* is the major cause of foodborne outbreaks linked to poultry eggs (Guard-Petter, 2001), while *Campylobacter* egg-associated outbreaks are extremely rare (Finch and Blake, 1985). A systematic review including a primary set of 4,316 references showed that *Campylobacter* was rarely isolated from the internal egg contents (Newell et al., 2011), which was also validated by several on-farm studies (Shanker et al., 1986; Van de Giessen et al., 1992; Pearson et al., 1993; Jacobs-Reitsma, 1995; Jacobs-Reitsma et al., 1995; Petersen et al., 2001; Smith et al., 2004; Callicott et al., 2006; Byrd et al., 2007; Kiess et al., 2007). Therefore, improving biosecurity systems and applying effective intervention strategies are the key elements to limit the prevalence of *Campylobacter* in broiler farms.

### Chicken Colonization of *Campylobacter*

Colonization of *Campylobacter* in farm chickens occurs usually due to horizontal transmission from the environment, such as *via* drinking water or animal feed. Once *Campylobacter* enters the chicken flock, it spreads rapidly and colonizes the intestinal tracts (crap, small intestine, and ceca) of most chickens after one week (Beery et al., 1988; Shanker et al., 1990; Newell et al., 2011). The level of *C. jejuni* inside these niches could be as high as 10^9^ cells/gram of intestinal tracts with no symptoms or noticeable harmful effects until slaughtering (Stern et al., 2001). One study reported that *C. jejuni* is not just a commensal bacterium in broiler chickens, but it can cause chronic inflammation, gut tissue damage, and diarrhea (Humphrey et al., 2014). In contrast, four combined and eight individual chicken genotypes showed no difference or negative effect on *C. jejuni* colonization and proliferation regardless of chicken growth rate or breed (Gormley et al., 2014).

Several factors affect chicken colonization by *Campylobacter*. These include chicken strain, *Campylobacter* strain, dosage of viable *Campylobacter* cells, and seasonality (Newell and Fearnley, 2003). Colonization potential of chickens by some *Campylobacter* strains could be enhanced by 1,000-folds (Ringoir and Korolik, 2003) or 10,000-folds (Cawthraw et al., 1996) under *in*-*vivo* experimental conditions, leading to the challenges to predict the ability of *Campylobacter* wild strains to colonize chicken flocks in the real commercial farms. There is generally a higher rate of colonization in summer than any other time of the year (Humphery et al., 1993). The colonization level (Wallace et al., 1997) and type of strains (Hudson et al., 1999) are also seasonally dependent. Besides high temperature and humidity, poultry houses require more ventilation during summer, which exposes the birds to more *Campylobacter* from the outside environment than any other time of the year (Hudson et al., 1999). Even individually caged birds showed a seasonal variation (increased to the peak in late April) in the fecal excretion of *C. jejuni*, suggesting that the surrounding temperature affects bird colonization even under limited conditions of *C. jejuni* transmission (Doyle, 1984).

Moreover, geographical locations, flock size, and type of the production systems (*i.e*., organic or conventional) can also influence the colonization of *Campylobacter* in chicken flocks (Newell and Fearnley, 2003). According to a previous study, up to 100% of flock were *Campylobacter*-positive in the case of organic and free-range flocks (Heuer et al., 2001). This is probably due to the exposure to the outside environment and a longer time the birds require to grow to the slaughter size compared to the indoor reared flocks. In the cases where the colonization of *Campylobacter* identified at species level, *C. jejuni* was the leading group by colonizing about 90% of *Campylobacter*-positive birds. The remaining ones were almost equally colonized by *C. coli* and *C. lari* (Uyttendaele et al., 1996). Several studies conducted in Europe suggested that the indoor-grown flocks were primarily colonized by one or two *C. jejuni* strains. Other studies conducted in North America and Australia showed that several *C. jejuni* strains usually colonized the indoor-grown flocks. This might be due to different levels of biosecurity standards in different countries as the incidences of *C. jejuni* colonization can be either due to the exposure to multiple sources consisting of different strains or a single source (*e.g*., feed or water) consisting of multiple strains. Interestingly, Hald and co-authors reported that *C. jejuni* colonization was higher in a total of 88 randomly selected poultry flocks raised in Danish farms that fed external grains compared to farms that fed home-grown grains (Hald et al., 2000).

Another important factor of chicken colonization is the adaptation capability and response of *Campylobacter* strains to the environmental conditions. For example, Gaynor and others identified a remarkable ability of *C. jejuni* to evolve rapidly during storage, culture, and condition passage (Gaynor et al., 2005). The colonization ability of *C. jejuni* 11168-O strain recognized as an excellent chicken colonizer was compared with *C. jejuni* 11168-GS clone recognized as a poor chicken colonizer after either aerobic or anaerobic incubation. The anaerobic priming of 11168-GS increased its colonization while the aerobic passaging of 11168-O decreased its colonization compared to their original strains.

Some procedures, such as feed withdrawal and transportation, affect the presence of *Campylobacter* in live chickens before their arrival into the poultry-processing plants. Feed withdrawal is a common commercial practice that the farmers remove the animal feeds from poultry houses 3 to 18 h before slaughtering (Byrd et al., 1998). The purpose of this practice is to clear the gastrointestinal tract and reduce the level of fecal materials in the body so as to minimize cross-contamination during poultry processing. Byrd and co-authors showed that feed withdrawal could increase the prevalence of *Campylobacter* in the crops of broiler chickens at the slaughter age (Byrd et al., 1998). *Campylobacter*-positive samples increased on average from 25% to 62.4% before and after feed withdrawal. The limitation of nutrients in the broiler crops might have resulted in a less diverse and competitive microbiota and subsequently enhanced the growth of *Campylobacter*. Transportation from farms to processing plants has been identified as a critical harbor for the transmission and colonization of *Campylobacter* in live birds. This is due to the reuse of contaminated crates for shipping, animal hoarding, and induced-stress during the transportation of live birds from different flocks and/or farms to slaughterhouses (Slader et al., 2002; Newell and Fearnley, 2003). Decontamination methods used for cleaning the reusable shipping crates for transportation was identified to be ineffective (Wedderkopp et al., 2001). Up to 70% *C. jejuni*-negative chickens became colonized after exposure to artificially contaminated shipping crates (Clark and Bueschkens, 1988). Whyte and others demonstrated that poultry overcrowding and stress induced during transportation could extensively increase the shedding of *Campylobacter* in fecal material of broilers and contributed to cross-contamination of their carcasses during processing (Whyte et al., 2001).

Several studies indicated that *C. jejuni* acts as a commensal and a super colonizer in chicken cecal microbiota (Awad et al., 2016; Connerton et al., 2018; Ijaz et al., 2018). Awad and co-authors identified that the microbial communities in the luminal and mucosa gut microbiome shifted in a timely manner during the growth of broiler chickens (Awad et al., 2016). A similar finding was observed when the chicken cecal microbiome was analyzed even without any artificial inoculation of *C. jejuni* (Ijaz et al., 2018). The critical periods for *C. jejuni* colonization ranged from 12 to 28 d of the broiler chicken age (Awad et al., 2016; Ijaz et al., 2018). Another report showed that *C. jejuni* appeared in 6-day old chicken birds (Connerton et al., 2018). The microbiota variation is usually influenced by the diet and microorganisms present in the surrounding environment, feed, and water (Connerton et al., 2018). For all of these reasons, it is quite challenging to inactivate *C. jejuni* once broiler chickens are colonized. Early prevention of *C. jejuni* colonization on poultry farms is very important to avoid further colonization. Although no symptom is associated with colonization of *C. jejuni* and *C. coli* in the broiler chickens, the high mortality rate might reflect the colonization prevalence (Powell et al., 2012).

### On-Farm Intervention Strategies Against *Campylobacter*

Early stage on-farm control of *Campylobacter* in broiler chickens has gained increasing attention during the last two decades because *Campylobacter* can effectively colonize chickens from the early days of their lives and remain prevalent at a high level throughout the poultry-processing line (**Table 2**). The potential of different intervention strategies by using vaccination, phage therapy, bacteriocins, probiotics, fatty acids, and essential oils has been investigated. Each strategy has some advantages and disadvantages (**Table 3**). For example, numerous bacteriocins (antimicrobial peptides) produced by commensal bacteria from chicken gut microbiota, such as *Lactobacillus salivarius*, could effectively inactivate *Campylobacter* under both *in-vitro* and *in-vivo* experimental settings (Svetoch and Stern, 2010). Specifically, the L-1077 bacteriocin was able to reduce >4 log CFU/g of *C. jejuni* in the cecal content. In a recent study, oral administration of three types of bacteriocins from *Lactobacillus salivarius* (OR-7) and *Enterococcus faecium* (E-760 and E50-52) were used in broiler chickens to investigate the development of resistance by *C. jejuni* (Mavri and Smole Možina, 2013). CmeABC multidrug efflux pump in *C. jejuni* played an important role in intrinsic and acquired resistance against bacteriocins. Thus, combining bacteriocins with an efflux pump inhibitor might synergistically inactivate *C. jejuni* and prevent the development of antimicrobial resistance.

**Table 2:** Examples of the average prevalence and load of *Campylobacter* throughout the poultry-processing chain.

Stage	Source	Prevalence (%) and/or average load of *Campylobacter*	Reference
Farm	Broilers	87.5%, 9 log CFU/g of cecal content (*n* = 50)	(Stern et al., 2001)
	Feces	96.4%, 5.16 log CFU/g of fecal content (*n* = 948)	(Stern and Robach, 2003)
Transportation	Caecum	6.5 log CFU/g of cecal content	(Achen et al., 1998)
	Feces	60–100% (*n* = 7 [10 flocks])	(Whyte et al., 2001)
Plant	Pre-scald	77%, > 6 log CFU/g of feather or skin (*n* = 40)	(Kotula and Pandya, 1995)
	Defeathering	3.9 log CFU/ml of carcasses rinse (*n* = 24)	(Berrang et al., 2000)
	Evisceration	96–100%, 2.7 log CFU/carcass (*n* = 48)	(Northcutt et al., 2003)
	Pre-chill	98%, 4.75 log CFU/ml of carcasses rinse (*n* = 450)	(Stern and Robach, 2003)
	Post-chill	84.7%, 3.03 log CFU/ml of carcasses rinse (*n* = 450)	(Stern and Robach, 2003)
	Pre-wash	87%, 4.78 log CFU/ml of carcasses rinse (*n* = 30 [4 processing plants])	(Bashor et al., 2004)
	Post-wash	80%, 4.30 log CFU/ml of carcasses rinse (*n* = 30 [4 processing plants])	(Bashor et al., 2004)
Retail		90%, > 4 log CFU/carcass (*n* = 552)	(Walker et al., 2019)

**Table 3:** Advantages and disadvantages of different prevention and control strategies against *Campylobacter* in poultry production.

Stage	Strategies	Advantage	Disadvantage
Farm	Vaccination	Preventive and promising (Annamalai et al., 2013; Neal-McKinney et al., 2014)	Expensive, highly specific, and difficult (Saxena et al., 2013; Kaakoush et al., 2015)
	Bacteriophages	Caused up to 5 log CFU/g reduction of *C. jejuni* in cecal content of commercial broiler flocks (Kittler et al., 2013)	Dilution in the gut over the time and development of resistance (Labrie et al., 2010; Fischer et al., 2013)
	Bacteriocins	Caused >4 log CFU/g reduction of *C. jejuni* under *in-vitro* settings (Svetoch and Stern, 2010)	Development of antimicrobial resistance by the multidrug efflux pump CmeABC (Mavri and Smole Možina, 2013)
	Probiotics	Part of the chicken gut microbiota (Kaakoush et al., 2015)	Limited reduction of *C. jejuni* after 15 d of oral administration (Santini et al., 2010)
	Short chain fatty acids	Ability to invade the gut epithelium cells (Davidson et al., 2005)	Limited reduction of *C. jejuni* under *in-vitro* settings (Davidson et al., 2005)

Several studies have investigated the efficacy of *Campylobacter* phages to either reduce *Campylobacter* count or prevent their colonization in chicken broilers (Carrillo et al., 2005; Wagenaar et al., 2005; El-Shibiny et al., 2009; Carvalho et al., 2010; Kittler et al., 2013). Some used artificial infections (Carrillo et al., 2005; Wagenaar et al., 2005; El-Shibiny et al., 2009; Kittler et al., 2013) while others used naturally infected birds (Carrillo et al., 2005). One study used an effective colonizer strain of *C. jejuni* and observed a significant reduction by several phages isolated from the same environment of the bacterial host (Wagenaar et al., 2005). Some phages caused up to 3 log reduction within the first 24 h, while others caused about 1 log reduction for up to 30 days at a high Multiplicity of Infection (MOI). The efficacy of *Campylobacter* phage therapy is not sufficient for a sustainable control of this bacterium. *C. jejuni* strain and phages used in that study were not isolated from representative samples of chicken farms, meats, or feces. Thus, this model cannot be generalized to be used in a wider therapeutic application in farms. However, the same study showed promising results and indicated that the high dose of phages (11 log PFU/ml) did not show any negative impact on the broilers’ health.

More studies are required to achieve sustainable benefit of *Campylobacter* phage therapy. The importance of using phages in the form of cocktail was observed in several studies (Wagenaar et al., 2005; El-Shibiny et al., 2009; Carvalho et al., 2010). Many other factors, such as oral administration route (*i.e*. phage delivery) and stability of individual phages, play important roles in the overall efficacy of phage therapy application in chicken broilers (Ushanov et al., 2020). For example, different studies indicated that the addition of phages into drinking water can be more effective than oral gavaging, which is not practical for large commercial scale production (Carrillo et al., 2005; Carvalho et al., 2010; Ushanov et al., 2020). However, such application requires more stable phages than others. Altogether, many studies agreed that phage therapy can be effective to reduce *C. jejuni* if administrated at a high MOI within 24 to 48 h prior to slaughter. Phage efficacy can also be improved when phages and hosts are isolated from the same environment. However, this may limit the application of phage therapy to specific poultry farm(s). Therefore, more phages and representative hosts need to be tested.

## *Campylobacter* in Poultry-Processing Plants

Poultry meat and eggs are important sources of dietary proteins, vitamins, and minerals. Poultry production is an intensively growing industry and chicken meat is one of the most produced meats around the world (Ritchie and Roser, 2017). The annual global amount of produced poultry meats has been rising by 10-folds within the last 50 years to approximately 102 million tons (Ritchie and Roser, 2017). Chicken is also one of the most sustainable major sources of dietary proteins as the feed conversion ratio (FCR; kg of feed/kg of edible weight) of chicken meat is only about 40% of the FCR of beef (Wilkinson, 2011). As a large, diverse and vertically integrated system involving animal farming and food processing, poultry production can be a common source of foodborne outbreaks. Either live poultry or poultry meat are important sources of *Campylobacter* and other important foodborne pathogens (Kaakoush et al., 2015). Both on-farm and *in vivo Campylobacter* controls are challenging due to the complexity and diversity of both systems (**Tables 2** and **3**). Alternatively, many studies have focused on controlling *Campylobacter* in the processing facilities.

### *Campylobacter* Survival During Poultry Processing

**Figure 2:**
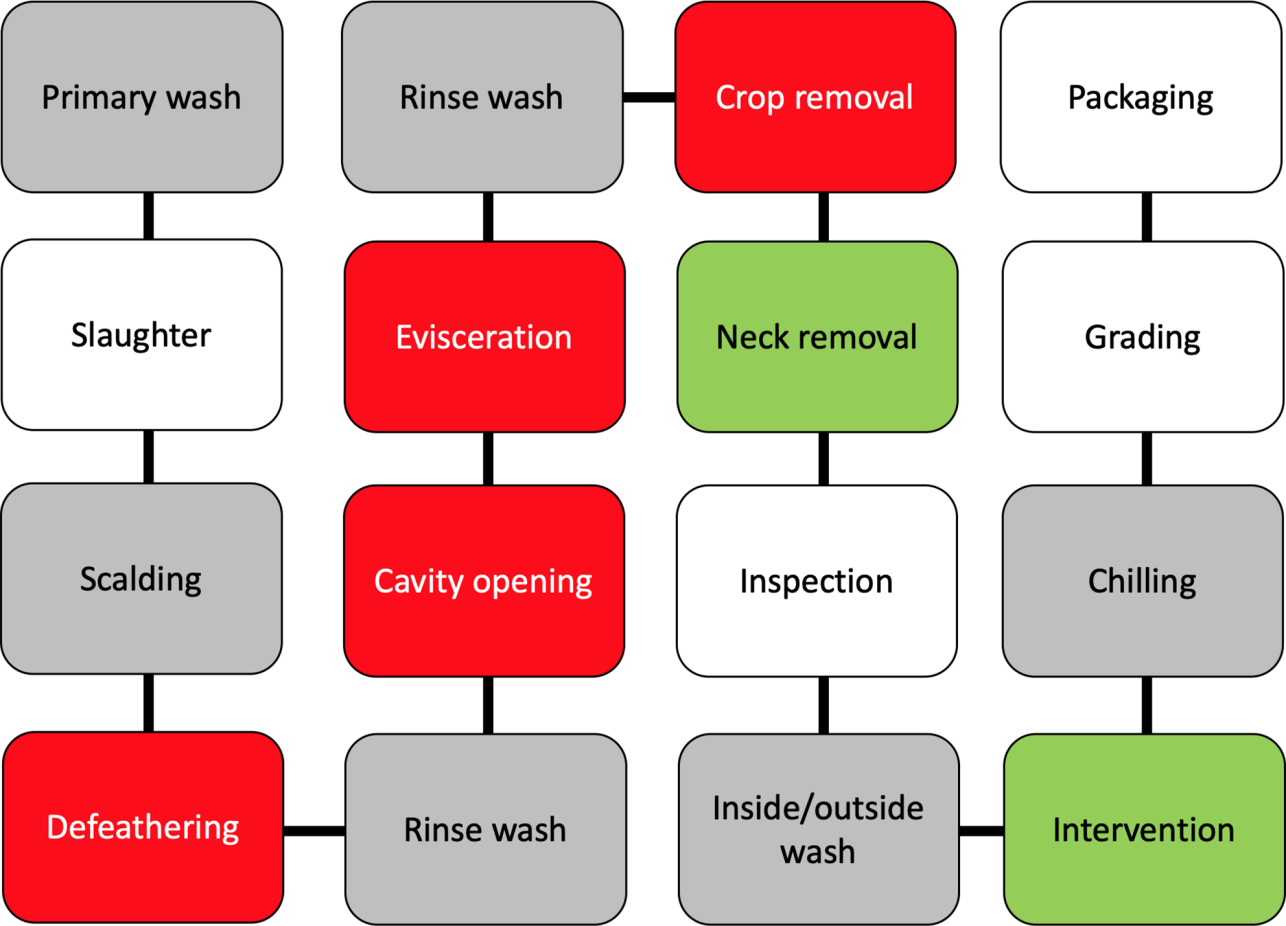
A schematic diagram of raw chicken processing steps. The gray boxes reflect steps that can enhance cross-contamination, the red boxes reflect steps where cross-contamination usually occurs, while green boxes reflect steps that contribute to the mitigation of *Campylobacter*.

Poultry processing is considered as an intensive procedure that requires highly trained personnel. One breach in either sanitation or hygiene practices might end in several cases of foodborne illnesses. *Campylobacter* enters a processing plant through any potentially contaminated bird(s) at an initial count as many as 10^9^ cells/g of cecal content (Beery et al., 1988; Stern et al., 2001). A single processing plant normally receives birds from multiple farms with variations in their ages, sizes, geographical locations, and production and biosecurity systems that increase the chance of *Campylobacter* contamination. Birds go through different processing steps starting from receiving and hanging until packaging. Processing consists of multiple critical points where *Campylobacter* starts to occur or increase in chicken carcasses (**Table 2**). Steps including scalding, defeathering, evisceration, nick removal, inside and outside (or inside-out) washing can all contribute to cross-contamination of *Campylobacter* in one way or another (**Figure 2**).

Scalding is a quick immersing of poultry carcasses into warm water (51–64°C) for a few seconds up to 2 min so as to loosen the skin follicles for defeathering. Berrang and others investigated the level of *Campylobacter*, total aerobic microbes, *E. coli*, and coliform throughout the poultry-processing plant (Berrang and Dickens, 2000). Total aerobic bacteria clearly decreased throughout the processing steps. In contrast, *Campylobacter* showed the highest recovery (increased from log 1.8 to 3.7 CFU/ml of chicken carcass rinse) compared to all the other bacteria after defeathering. Heating of poultry carcasses followed by chilling during the processing steps are essential in assisting practices for effective defeathering. However, this temperature fluctuation creates several challenges to control microorganisms including *Campylobacter*. For example, skin follicles remain open after scalding that allows bacteria to penetrate the skin and accumulate inside the follicles. Moreover, the follicles close again during chilling, making the poultry decontamination to be highly challenging. In addition, a large shift appears in the native skin microbiome of chicken (Thomas and McMeekin, 1980). The predominately Gram-positive skin microbiota (*e.g*., *Micrococcus*) is usually detached and replaced by a population mixture consisting of a majority of Gram-negative bacteria. However, the alteration of chicken meat microbiome during processing varies based on multiple factors, including geographical location, season, and bird-to-bird. One common factor is that scalding liquidizes some fats on the skin that became part of chicken juice and other surrounding fluids. The liquid fat solidifies again during the chilling step and creates a lipid film on the surface of chicken meat. Both scanning electron microscopy and transmission electron microscopy showed that scalding and defeathering scraped off the epidermis cells of chicken skin that became smoother and less hydrophobic than normal after processing (Thomas and McMeekin, 1980). The bacterial contaminants were identified within a protective fluid film formed both on the surface and inside deep channels of chicken skin after chilling, which makes microorganisms in chicken meat unapproachable by the antimicrobial agents.

Moreover, evisceration is one of the most critical steps of cross-contamination. Colonized gastrointestinal tract of poultry birds carries a large number of *Campylobacter* bacteria that can spread in a wide range, especially in the case of gut leakage. Many in-plant studies confirmed that the number of *Campylobacter*-positive carcasses significantly increased after this process (Berrang et al., 2001; Northcutt et al., 2003; Keener et al., 2004). For example, *Campylobacter*-positive chicken thighs and breasts separately increased from 0% to 90% at a level between 2 and 3 log CFU/g after evisceration (Berrang et al., 2001). Another study identified that *Campylobacter* contamination level was higher on the breast meat than the thigh meat or drumstick (Kotula and Pandya, 1995). Leaking of *Campylobacter* from the gut during evisceration can contaminate the lower half of the carcasses (breast and neck) more than the upper half (thighs and drumstick) as the birds are always hanged upside-down by the feet. The hanging necks of carcasses were also frequently determined to be *Campylobacter*-positive (Kotula and Pandya, 1995).

Poultry carcasses require rapid cooling to prevent the growth of microorganisms after evisceration. Chilling and antimicrobial treatment are usually combined in many processing plants to save energy and rapidly inhibit bacterial growth by washing the carcasses with cold chlorinated water (Keener et al., 2004). Poultry carcasses are usually washed by dipping or spraying using chlorinated water to remove blood, tissue, fragments, and contamination after evisceration. Dipping can cross-contaminate carcasses under commercial processing conditions especially when processing a large number of birds at the time (Bailey et al., 1987; Bilgili et al., 2002; Demirok et al., 2013). In contrast, spray washers tend to reduce the level of cross-contamination on the chicken meat (Keener et al., 2004; Demirok et al., 2013). Several options of spraying systems for poultry carcasses have been used in poultry industry, including brush, cabinet, and inside-out washing systems (Keener et al., 2004). The brush washing system is similar to a car washer where many rubber fingers are used with the aid of water to remove debris and wash the carcasses from the outside. The cabinet washing system contains multiple sprayers in an enclosed system to wash the outside of the carcasses. Inside-out system is a similar enclosed spraying system but used for both external and internal washing at the same time. The machine rotates the carcasses and sprays them from the outside, while probes of single sprayers enter the intestinal cavities of carcasses to wash them from the inside. Many inside-out washing machines spray water at the pressure level between 40 and 180 psi to remove visible fecal contamination and fragments (Keener et al., 2004). Chlorine concentration ranged from 20 to 50 mg/L and water consumption ranged from 100 to 200 L/min.

### Antimicrobial Treatments for Poultry Processing

Many laboratory-scale experiments showed that the approved antimicrobials such as acidified sodium, chlorite, cetylpyridinium, chlorine, chlorine dioxide, peroxyacetic acid, and trisodium phosphate could cause up to 5 log reduction of *Campylobacter* in chicken meat (**Table 3**). However, in-plant poultry washers have limited effect on inactivating *Campylobacter* in chicken meat regardless of the efficacy of antimicrobials, water temperature, or washing system. This could be due to several factors including the presence of large molecules in chicken meat (*e.g.*, proteins and lipids) and *Campylobacter* in chicken skin due to changes induced by processing, sensitivity of chicken skin to heat, oxidation and discoloration, initial microbial load of carcasses, number of processed carcasses per min, interaction or masking of antimicrobials (*e.g*., chlorine) by organic materials in the processing water, water quality and survival of *Campylobacter* in recycled processing water, poor sanitation, accumulation of lipids, fecal materials, and/or organics at any point through the processing line. It is worth mentioning that there is no effective critical control (*i.e*., killing) point in processing raw chicken similar to that of the pasteurization step for milk processing (Tresse et al., 2017).

### Current Situation of Raw Poultry Product Safety

The prevalence of *Campylobacter* in poultry products is clearly a major food safety challenge for many years. It is important to target chicken as a critical food vehicle of *C. jejuni* due to the high rate of contamination. More on-farm and in-plant control strategies became available in the recent years, but these strategies need improvement to enable effective inactivation of *Campylobacter* at an early stage or in chicken end-products. In 2015, the United State Department of Agriculture, the Food Safety and Inspection Service agency (USDA-FSIS) established a new *Campylobacter* and *Salmonella* performance standard for the contaminated poultry products, raw chicken parts (*e.g*., breasts, thighs, wings), and not ready-to-eat (NRTE) poultry products (Crim et al., 2015). For example, 8 out of 51 *Campylobacter*-positive broiler carcasses is the maximum acceptable number of randomly tested samples. In 2018, new antimicrobial agents have been approved by the USDA-FSIS to be used in washing poultry carcasses during processing. These include peroxyacetic acid (a mixture of hydrogen peroxide and acetic acid), a mixture of calcium chloride, calcium hypochlorite, sodium chloride, calcium hydroxide, calcium carbonate, sodium triphosphate, and a combination of calcium chloride with sodium bisulfate (Service, 2018). Although *Campylobacter* can be reduced to some extent by antimicrobials, they still might not be reduced to a safe level as only a few hundred cells might cause human illnesses (Black et al., 1988; Hara-Kudo and Takatori, 2011). In addition, in-plant antimicrobial treatment requires intensive amount of water to wash chicken carcasses. For example, a medium size poultry-processing plant spends annually $0.5 to 1 million USD on average on water consumption for washing chicken carcasses and surfaces (Jackson, 1999), but *Campylobacter* reduction is still insufficient.

## Alternative Strategies to Control *C. jejuni* in Agri-Foods

### Plant-Based Antimicrobials

Plant-derived compounds have been used for centuries in medicine, perfumery, cosmetics or being added to foods as oils, herbs or spices (Hyldgaard et al., 2012). For example, herbs and essential oils were initially used in medicine due to their antimicrobial, anti-inflammatory, or antioxidant effects, then their application expanded in agri-foods in the 19^th^ century for their aroma and flavors. These antimicrobials are important secondary metabolites that play major roles in plant defense systems to protect them from microbial infections (Tajkarimi et al., 2010). It was estimated that ~3,000 essential oils have been identified and ~300 are commercially available for flavoring, fragments, or cosmetics (Van de Braak and Leijten, 1999). In addition, essential oils can act as growth promoters in farm animals similar to antibiotics (Brenes and Roura, 2010; Ahmadifar et al., 2011). A histology study showed that feeding different plant extracts to chicken broilers increased the thickness of the mucus layer in the glandular stomach and jejunum (Jamroz et al., 2006). These changes were associated with a large shift in gut microbiota that could hypothetically promote the growth of birds.

Cinnamon is one of the earliest spices used in human history and cinnamon oil is among the most studied essential oils due to its high antimicrobial potency (Ravindran et al., 2003). The genus *Cinnamomum* consists of ~250 different species. *C. verum* and *C. cassia* are the most known and used herbal medicines or spices. These plants are the main natural sources of cinnamon. Cinnamon oil consists of several major antimicrobial compounds, including cinnamaldehyde (70–90%), 1-linalool, p-cymene, and eugenol (Davidson et al., 2005). Aldehyde groups are reactive organic compounds that can crosslink covalently with proteins and nucleic acids through amine groups. Therefore, the mode of action of cinnamaldehyde is inconclusive. Several mechanisms can occur depending on the bioavailability and concentration of the system (Hyldgaard et al., 2012). For example, cytokinesis can be inhibited due to the inhibition of different enzymes by cinnamaldehyde at a low concentration. ATPase inhibition occurs at the sub-lethal concentration, while the alteration of fatty acid composition of cell membrane, cell leakage and cell death occur at the lethal concentration.

In comparison, curcumin is the major active compound of the rhizome of turmeric (*Curcuma longa*). This golden spice is a phenolic pigment responsible for the yellow color of turmeric. Numerous studies have shown that curcumin can effectively inactivate both Gram-negative and Gram-positive bacteria (Rudrappa and Bais, 2008; Kaur et al., 2010; Tyagi et al., 2015). However, limited studies have investigated the antimicrobial mechanism of curcumin. Blocking the assembly of the FtsZ protein essential for forming the FtsZ ring (*i.e.*, Z ring) to initiate cell division in bacteria was identified to be the mode of action against *Bacillus subtilis* and *E. coli* (Kaur et al., 2010). In contrast, curcumin has been found to attenuate several virulence factors, including quorum sensing and biofilm formation in *P. aeruginosa* (Rudrappa and Bais, 2008). A recent study examined the membrane permeability of *S. aureus, Enterococcus faecalis*, *E. coli*, and *P. aeruginosa* after being treated with curcumin (Tyagi et al., 2015). A steady-state fluorescence and flow cytometry analyses showed uptake in the extracellular propidium iodide (only enters intact bacterial cells by a permeabilizing agent) and leakage of calcein (only leak out of bacterial cells if there is membrane damage due to cell wall membrane damage) in both Gram-positive Gram-negative bacteria. Antimicrobial mechanism of curcumin is different depending on the bacteria studied and the assays used (Han et al., 2006; De et al., 2009; Kaur et al., 2010; Tyagi et al., 2015). More studies are still needed to confirm the antimicrobial mechanism(s) of the action of curcumin.

To the best of our knowledge, the specific antimicrobial mechanism of curcumin against *Campylobacter* has not been investigated. The effect of curcumin against *Helicobacter pylori*, a highly relevant bacterium to *Campylobacter*, has been repeatedly confirmed in several studies (Di Mario et al., 2007; Zaidi et al., 2009; Sarkar et al., 2016; Vetvicka et al., 2016). One study of using a high-throughput screening of 5,000 chemical compounds discovered that the inhibition of *H. pylori* by curcumin was due to the inhibition of shikimate pathway (Han et al., 2006). This pathway is essential for the synthesis of aromatic amino acids (*e.g*., phenylalanine, tryptophan, and tyrosine) in bacteria, fungi, and higher plants, but not in mammals. Targeting this particular pathway makes curcumin a very safe antimicrobial agent for human consumption. In fact, curcumin showed no toxicity on human health even used at a level as high as 8,000 mg per day (Cheng et al., 2001; Lao et al., 2006). In addition, the antimicrobial activity of curcumin against *H. pylori in vitro* (65 clinical isolates) and *in vivo* during infections in mice were examined. The minimum inhibitory concentration (MIC) of curcumin ranged from 5 to 50 μg/ml regardless of genetic variation of the tested *Helicobacter* strains. Curcumin not only inactivated *H. pylori* during infection but also reduced the gastric damage induced by *H. pylori* infection to almost a normal state. Although limited studies have identified the antimicrobial mechanism of curcumin, available evidence shows its great potential for preventing and treating bacterial contaminations and infections.

### Metal Oxide Nanoparticles

Novel applications of nanotechnology and nanomaterials have gained great attention in the recent years. For example, the applications of metal oxide nanoparticles (*e.g.*, Al_2_O_3_, TiO_2_, and ZnO NPs) could inactivate several foodborne pathogens in a variety of agri-food systems (Fernández et al., 2009; Akbar and Anal, 2014; Panea et al., 2014). ZnO was identified to be more effective than other metal oxides (*e.g.*, CuO and Fe_2_O_3_) against both Gram-negative and Gram-positive bacteria (Azam et al., 2012). In addition, ZnO NPs was more effective against *C. jejuni* than other Gram-negative bacteria including *E. coli* O157:H7 and *S. enterica* (Xie et al., 2011). The direct contact of ZnO NPs (positively charged) with bacterial cell wall (negatively charged) by electrostatic force leads to destabilization and disruption of bacterial outer cell membrane. In addition, semi-conductive property of ZnO allows the generation of reactive oxygen species that can attack different cytoplasmic and extra-cytoplasmic targets after the binding (Sirelkhatim et al., 2015).

### Synergism

Antimicrobial combinations have been used since the earliest days of the recorded history to treat illnesses and reduce sufferings (Chou, 2006). Therapeutic use of traditional Chinese herbs is a prime example. Indeed, antibiotic is one of the most important drug discoveries in the modern medicine. However, the emergence of antibiotic resistance to most available antibiotics became a serious public health concern in the recent years and near future (de Kraker et al., 2016). A synergistic combination of antimicrobials can minimize some of the disadvantages associated with the use of antimicrobials, such as the development of bacterial resistance, high dosage, and limited effect (Chou, 2006). Antimicrobial combination has been extensively studied to inactivate some highly challenging bacterial and viral infections including methicillin-resistant *Staphylococcus aureus* infection (An et al., 2011) and human immunodeficiency virus infection (Gaibani et al., 2019).

Synergism is defined as an effect that is greater than the sum of multiple individual effects. Many approaches, hypotheses, methodologies, and models have been used to study the synergism in different fields, including microbiology, pharmacology and enzymology (Chou, 2006). The definition of synergism is a very controversial topic due to the complexity of biological systems and some possible mathematical errors or pitfalls in the combinatorial studies. Some important concepts such as the difference between synergism and enhancement or potentiation are not fully clear. For example, if antimicrobial A has a quantifiable effect (*e.g*., 10%), while antimicrobial B has no effect (*i.e.*, 0%), and their combination produces an effect greater than antimicrobial A (*e.g*., 20%), then this is considered as an enhancement or potentiation, but not a synergistic interaction. In contrast, synergism is an effect greater than the sum of multiple quantifiable effects (*e*.*g.*, 10% + 10% = 30%). In addition, the additive effect has always to be less than 100%. For example, if antimicrobial A and B each affects 20%, the additive effect is not simply 40% because if each antimicrobial produces 70% effect the combined effect cannot be 140%. Chou and Talalay reported the fractional product equation to solve this issue [(1–0.7) (1–0.7) = 0.09] where the additive effect can never exceed 100% (Chou and Talalay, 1984).

### Methods for Identifying Antimicrobial Synergism

Three methods are most used in antimicrobial combination studies. These include the disk diffusion method, time killing method and fractional inhibitory concentration index method (FICI) (Odds, 2003; Zhou et al., 2016). Disk diffusion method is a simple visual test that relies on comparing bacterial inhibition zones of diffused (single and combined) antimicrobial agents in the agar plates. Time killing method shows how a bacterial population responds to the antimicrobial treatment at different time intervals in either broth or agar medium. It relies on monitoring the antimicrobial effect of single and combined antimicrobials by calculating the log reduction of lethal and sub-lethal concentrations over time. For example, if antimicrobial A caused 1 log reduction and antimicrobial B caused 1 log reduction, then the additive effect would be 2 log reduction. In this case, synergism would require effect greater than 2 log reduction (*e.g*., 1 + 1 = 3). This method is labor-intensive and time-consuming. Thus, a few concentrations of antimicrobials are usually used and combined at a fixed ratio. In contrast, the FICI method (also called microdilution checkerboard) shows a clear visualization of positive/negative inhibitory interactions of multiple ratios of combined antimicrobials. It relies on constructing two antimicrobial combinations in a two-dimension array (*e.g.*, 96-well plate) and comparing the MICs of single and combined treatments. Synergy requires at least a four-fold reduction in the MIC of both antimicrobials combined (*i.e.*, FICI value of ≤ 0.5). The FICI method gained more popularity in the recent years as it is more restricted in identifying synergism, more comprehensive, and easier to construct and interpret than other methods.

### Types and Mechanisms of Antimicrobial Synergism

Different types of antimicrobial interactions can occur between antimicrobials depending on their origins and individual mechanisms. It is common to observe synergism between antimicrobials of different mechanisms and different targets (Jia et al., 2009; Oh et al., 2015). For example, combining efflux pump inhibitor(s) with an intracellular antimicrobial(s) can synergize to inactivate microorganisms that use efflux pumps to remove antimicrobials due to antimicrobial accumulation inside the cells (Oh et al., 2015). In addition, antimicrobials may synergize due to the complementary or facilitating collective actions (Jia et al., 2009). Although different antimicrobials may have different targets and mechanisms, they might have overlapping pathways at the molecular level. More importantly, synergism can be used to increase bacterial antibiotic susceptibility.

### Applications of Synergism

Bacteria develop resistance to antibiotics *via* different mechanisms (Mavri and Smole Možina, 2013). These include the modification of a receptor or active site of the antibiotic target to prevent or reduce binding, production of enzymes that directly destruct or modify the antibiotics, and/or reducing the accumulation inside the cells by decreasing the outer cell membrane permeability or pumping out the antibiotics using efflux pumps. One of the best applications for antimicrobial synergism is to be against tolerant and/or resistant pathogens that require more than single or additive treatments. For example, Augmetin^®^ is a common commercial antibiotic that consists of a combination of clavulanate acid and amoxicillin to inactivate different pathogens, including *β*-lactam resistant bacteria (12). The combination of clarithromycin and amoxicillin is part of the standard therapy for *H. pylori* stomach infections (11).

Plant-based antimicrobials are a great source of new alternative antimicrobials. Many recent studies showed that plant-based antimicrobials (*e.g*., phenolic compounds) synergize with antibiotics (*e.g*., amikacin, ceftriaxone, cephradine, methicillin, imipenem) (Oh and Jeon, 2015) or metal oxide nanoparticles (Hemaiswarya and Doble, 2010) against various microbes. Oh and Jeon reported synergistic interactions of several phenolic compounds (*e.g*., gallic acid and taxifolin) in combination with ciprofloxacin or erythromycin against fluoroquinolones- and macrolides-resistant *C. jejuni* isolates (Oh and Jeon, 2015). Phenolic compounds increased membrane permeability as determined by measuring the intracellular uptake of 1-*N*-phenylethylamine. As a result, accumulation of both antibiotics increased substantially inside the bacterial cells. Further testing showed that phenolic compounds increased 1-*N*-phenylethylamine accumulation in an isogenic (knockout) *cmeB* mutant more than that in a wild type *C. jejuni* strain. In addition, the expression level of CmeABC multidrug efflux pump was reduced by several phenolic compounds (*i.e*., gallic acid and taxifolin). These findings indicated that phenolic compounds increased the influx rate and decreased the efflux rate of antibiotics.

### Active Packaging

Food packaging is one of the last steps in food processing to ensure that the food products are contained and delivered in the best condition. Packaging materials and/or the atmospheric condition inside the packaging are used to protect the foods from microbial growth, pathogen contamination, physical damage, chemical degradation, or other effects from the environment. Most of the commercially applied packaging technologies aim to preserve food quality and extend the shelf life of food products. Moreover, data regarding the use of active packaging to control foodborne pathogens in potentially contaminated and high-risk foods is still limited compared to those for spoilage bacteria. For example, modified atmospheric packaging (MAP) was extensively studied for the control of spoilage microorganisms in a variety of food products, such as raw meats, fresh produce, and seafood products (McMillin, 2008). In addition, recent technologies and intervention strategies that are used in food processing allow food packaging to be a suitable component of hurdle technology. Such approach might overcome the challenge of controlling the survival of frequently isolated pathogens from commercially available raw meats, such as *C. jejuni* in chicken, *V. parahaemolyticus* in seafood, and pathogenic *E. coli* in beef.

Fresh poultry, raw meats, and seafoods are considered as high-risk and highly perishable foods. Foods of animal origins including raw milk, raw cheese, and raw meats have a high content of moisture and nutrients. These factors form an ideal environment for rapid growth and/or long survival of many microorganisms, including both spoilage and pathogenic bacteria. Different bacteria have been commonly isolated from fresh chicken meats after processing. These include *Micrococcus*, Gram-positive rods*, Cytophaga-Flavobacterium*, *Pseudomonas*, and Enterobacteriaceae (Thomas and McMeekin, 1980). Thomas and McMeekin identified that poultry carcasses originally carried *Micrococcus* as a part of the skin microbiome, but contamination with the psychrotrophic *Pseudomonas* appeared after processing (Thomas and McMeekin, 1980). A whole-genome sequencing analysis showed that the Firmicutes (mainly Gram-positive) were the most abundant bacterial group based on the phyla level of raw chicken meat after processing (Kim et al., 2017).

*Campylobacter* and *Salmonella* are the most frequently isolated human pathogens from poultry products at the retail level (Rouger et al., 2017). No correlation was established between the prevalence of these two poultry-associated pathogens in chicken carcasses collected from 58 slaughterhouses during a 12-month period in France (Hue et al., 2011). Moreover, no correlation was established between the microbial load (*i.e*., total aerobic count, Enterobacteriaceae, and coliform) and the prevalence of *Campylobacter* in chicken and turkey fresh meat cuts (Fontanot et al., 2014). Thus, the prevalence of *Campylobacter* in poultry end products is random and unpredictable because transmission in farms and cross-contamination during processing can occur at any point, which is not always associated with any other microbial indicators. The large size of poultry industry and production scale makes the detection of *Campylobacter* more challenging in these products. Both *Campylobacter* and *Salmonella* can originally occur at a high level (up to 10^8^ CFU/g) in the gastrointestinal tract of birds, but their prevalence in poultry meat varies depending on the cross-contamination incidents. A positive correlation was identified between *Campylobacter* level in chicken caeca and the end products (Hue et al., 2011). *C. jejuni* and *C. coli* were equally prevalent in chicken caeca, but *C. jejuni* was the most frequently isolated one from processed carcasses. These findings highlight the difficulty of preventing the presence of *C. jejuni* in poultry end products from *Campylobacter*-positive carcasses.

Bioactive packaging is an effective method for the control of common foodborne pathogens in foods, including raw meat and fresh produce. It can be used for quality preservation purposes to limit the growth of spoilage microflora, or reduce the prevalence of foodborne pathogens in high-risk foods (Panea et al., 2014; Hakeem et al., 2020). For example, different coliphages and *Listeria* phages were immobilized on active packaging materials to control *E. coli* O104:H4 in alfalfa sprouts during germination and *L. monocytogenes* in cantaloupes during cold storage (Lone et al., 2016). One major issue is that most phages are not stable under dehydration condition, which limit their applications in bioactive packaging (Anany et al., 2011). Thus, many studies have used phages in the absorbing pads that are usually placed under fresh foods to absorb moisture and fluids. This can maintain the quality and freshness of fresh foods and protect phages from desiccation at the same time (Hakeem et al., 2020).

## Concluding Remarks

*Campylobacter* is one of the leading foodborne pathogens responsible for human gastroenteritis. No effective control method is available to prevent *Campylobacter* contamination either in poultry farms or poultry-processing plants. Both poultry farms and processing systems are complex and require intensive operations. In addition, the use of antibiotics as growth promoters was banned to limit antibiotic resistance in different countries. The efficacy of many approved antimicrobials on the reduction of *Campylobacter* in poultry-processing plants is limited. For all of these aforementioned factors, new generations of antimicrobials including novel synergistic antimicrobial combination are required for *Campylobacter* control and prevention in the agro-ecosystem. Considering the challenges in controlling *C. jejuni* in poultry farms and processing plants, innovative antimicrobial packaging to reduce *C. jejuni* in raw chicken meat at the retail level is needed. Many new effective approaches are available for the control *C. jejuni* to enhance the safety of the end products. These include bioactive packaging of bacteriophages or nanoparticles as well as the use of synergistic combinations of antimicrobials to maximize the advantages and minimize disadvantages associated with their uses.

## Author Contributions

MH wrote the manuscript. XL supervised, reviewed, and edited the manuscript. All authors contributed to the article and approved the submitted version.

## Funding

XL was supported by Genome British Columbia/Genome Canada [SIP021] and the Natural Sciences and Engineering Research Council of Canada in the form of Discovery Grant (NSERC RGPIN-2019-03960) and Discovery Accelerator Grant (PGPAS-2019-00024). MH received a 5-year scholarship (2015-2020) from King Saud University.

## Conflict of Interest

The authors declare that the research was conducted in the absence of any commercial or financial relationships that could be construed as a potential conflict of interest.
